# Breast Cancer Patterns in Saudi Arabia (2007–2022): A Nationwide Cancer Registry Surveillance Study

**DOI:** 10.3390/jcm15103983

**Published:** 2026-05-21

**Authors:** Nuha Alsaleh, Shatha Alduraywish, Maria A. Arafah, Shaima Ali Maghdi, Mohamed Alghamdi, Tamrah Alrammah

**Affiliations:** 1 Department of Surgery, King Saud University, Riyadh 12355, Saudi Arabia; shaimamaghdi@gmail.com; 2 Department of Family and Community Medicine, King Saud University, Riyadh 12355, Saudi Arabia; salduraywish@ksu.edu.sa; 3 Department of Pathology, College of Medicine, King Saud University, Riyadh 12355, Saudi Arabia; marafah83@gmail.com; 4 Department of Surgical Oncology, Jazan Specialized Hospital, Jazan Health Cluster, Jazan 82513, Saudi Arabia; 5 Oncology Center, King Khalid Medical City, Riyadh 11535, Saudi Arabia; alghamditbcc@gmail.com; 6 Department of General Surgery, Diriyah Hospital, Riyadh Third Health Cluster, Ministry of Health, Riyadh 12372, Saudi Arabia; alrammaht@gmail.com

**Keywords:** breast cancer, cancer registry, epidemiology, stage at diagnosis, Saudi Arabia

## Abstract

**Background:** Population-based cancer registry surveillance is essential for monitoring breast cancer burden and guiding cancer control planning; however, national surveillance evidence from Saudi Arabia remains limited. Using the Saudi Cancer Registry (SCR), we describe the distribution of age at diagnosis, geographic location, registry stage, histology, and grade among Saudi women diagnosed with breast cancer between 2007 and 2022. **Methods:** We performed a retrospective descriptive study of all Saudi female breast cancer cases registered in the SCR from 1 January 2007 to 31 December 2022 (*N* = 40,755). Variables were coded according to SEER guidelines; STATA 16 was used for analyses. **Results:** The average age at diagnosis among 40,755 cases was 51.85 years. The highest case volume was from Makkah (25.5%), Riyadh (23.6%), and the Eastern Province (15.9%). The national age-standardized incidence rate (ASR) increased from 18.2 to 49.7 per 100,000 women between 2007 and 2022. Invasive ductal carcinoma (no special type) was the most common histology (76.7%). Overall, 42.7% of cases were localized, 36.5% regional, 14.2% distant, and 6.6% unstaged. Stage distribution differed significantly by age group (χ^2^  =  98.1, *p* < 0.001) and by region (χ^2^  =  312.6, *p* < 0.001). **Conclusions:** National cancer registry data show marked regional differences in breast cancer incidence and a persistent proportion of late-stage diagnoses. These findings may inform early detection planning and region-specific cancer control strategies.

## 1. Introduction

The most commonly diagnosed female cancer in the world is breast cancer, which is a prominent cause of cancer morbidity and mortality. Although survival and detection rates have improved in many high-income countries, there is still wide variation in detection by age, stage at diagnosis, and burden by geographical area, which is partly explained by differences in population structure, screening policies, and health system capacity [1]. One of the main ways these patterns are systematically recorded is through population-based cancer registries, which can generate reliable estimates of cancer burden over time and inform the planning of cancer control activities at the national level [2].

In Saudi Arabia, breast cancer is the leading reported cancer among women and is a large public health issue. Data from the National Registry show a continuous rise in reported incidence in recent decades, in parallel with rapid demographic shifts, urbanization, and greater access to health services [3]. Although studies exploring certain properties of breast cancer in institutional or regional samples exist, there is little recent research that adequately addresses national trends and long-term patterns [4,5]. National registry surveillance is therefore essential to monitor stage at diagnosis and regional disparities, and to guide cancer control and early detection strategies.

The Saudi Cancer Registry (SCR) was established to document and monitor the occurrence of cancer in all administrative districts in the country. Descriptive data of the cancer registries reports information on various demographic features, as well as on the tumor morphology, histological grade, and summary stage at diagnosis, all of which are coded following standardized manual instructions for classification, so that data can be efficiently compared to that of other regions or time periods [3]. While registry data lack information on molecular subtype, treatment, and outcomes, they do offer strong potential as a surveillance system for the burden of cancer and diagnostic patterns at the population level.

Age distribution, regional distribution, histological pattern, and stage at diagnosis are important surveillance indicators for cancer control planning. In the literature, stage at diagnosis has been associated with factors such as disease awareness, screening participation, and access to diagnostic services, although causal relationships cannot be inferred from registry data alone [6]. This population-based registry surveillance study uses Saudi Cancer Registry data to characterize breast cancer diagnosis patterns among Saudi women from 2007 to 2022, aiming to support cancer control planning and inform region-specific interventions.

## 2. Methods

### 2.1. Study Design and Setting

This retrospective, population-based cancer registry surveillance study used data from the Saudi Cancer Registry (SCR) to describe national breast cancer diagnosis patterns in Saudi Arabia from 2007 to 2022. The SCR was created in 1992 as a national registry under the National Registries Department of the Saudi Health Council, Ministry of Health, Saudi Arabia. Cancer is a notifiable disease in the Kingdom, and all cancer cases must be registered at the national level in governmental, private, and military hospitals, clinics, and laboratories across all administrative provinces of the country.

### 2.2. Study Population

The total sample size was 40,755 Saudi women who were diagnosed with primary breast cancer from 1 January 2007 to 31 December 2022. Cases were defined by topography codes ranging from C50.0 to C50.9 in the International Classification of Diseases for Oncology, Third Edition (ICD-O-3). All non-Saudi patients and records with missing data with regard to sex and year of diagnosis were excluded. The SCR records Saudi and non-Saudi cases separately; this analysis was restricted to the Saudi national population to produce nationally representative estimates. Duplicate records were identified and removed using SEER multiple-primary rules (same patient, same site, same histology within two months) prior to analysis.

### 2.3. Data Collection and Registry Scope

Cancer data were obtained from medical records and abstracted by SCR-certified tumor registrars only for those patients with clinical and/or histopathological evidence. Information on demographic characteristics, age at diagnosis, region of residence, tumor morphology, histological grade, and stage at diagnosis was consistently collected and coded using the SCR’s standardized coding structures. Tumor grade, histology, and stage were categorized according to SEER standards. Registry coverage has been stable across all years and regions of the study period, as confirmed by published SCR annual reports [3]. Molecular subtype, treatment, recurrence, and survival data are not captured by the SCR.

### 2.4. Statistical Analysis

The SCR supplied data in Microsoft Excel (Microsoft Corporation, Redmond, WA, USA) format, and these datasets were checked and cleaned prior to analysis. All statistical analyses were conducted in STATA 16 software (StataCorp, College Station, TX, USA; RRID: SCR_012763). Descriptive statistics, including means and standard deviations for continuous variables and frequencies and percentages for categorical variables, were used to summarize the data. Age-standardized incidence rates (ASRs) were calculated using the World Standard Population (WSP). Chi-square tests were used to assess associations between age group and SEER summary stage, and between region and SEER summary stage; results are presented with test statistics, degrees of freedom, and *p*-values. A *p*-value of <0.05 was considered statistically significant. Multivariable logistic regression was not performed, as the SCR dataset does not provide individual patient-level records with the full covariate structure required for valid regression modeling. ASR temporal trend data were derived from published annual SCR incidence reports [3,4,7].

### 2.5. Variable Definitions

The key study variables were defined as follows. Age at diagnosis was recorded in years at the time of first histopathological or clinical diagnosis in the SCR, and was grouped as <30, 30–39, 40–49, 50–59, 60–69, and ≥70 years. Region of residence was the administrative region at time of diagnosis across the 13 Saudi administrative regions. SEER summary stage was coded as localized (confined to the breast), regional (spread to regional lymph nodes or adjacent tissue), distant (metastatic), or unstaged (insufficient information). Histological subtype was classified per ICD-O-3. Tumor grade was coded as Grade I–IV or unknown. The ASR was calculated using the direct method with the WSP, expressed per 100,000 women. Breast cancer topography was defined using ICD-O-3 codes C50.0–C50.9.

### 2.6. Ethical Considerations

This study was approved by the Institutional Review Board, King Saud University Medical City, King Saud University—College of Medicine, Riyadh, Saudi Arabia (Research Project No. E-25-9595; Ref. No. 25/0386/IRB; Approval date: 4 May 2025). The study utilized de-identified secondary registry data and did not require individual informed consent.

## 3. Results

### 3.1. Patient Characteristics, Age Distribution, and Geographic Distribution

**Figure 1:**
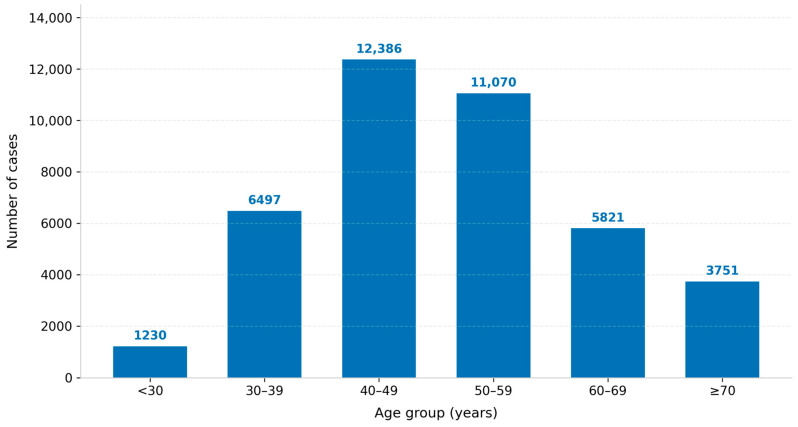
Age Distribution of Breast Cancer Cases Among Saudi Women Diagnosed Between 2007 and 2022.

A total of 40,755 Saudi female breast cancer cases were registered in the Saudi Cancer Registry between 2007 and 2022. The mean age at diagnosis was 51.85 years. Figure 1 shows that the largest proportion of cases occurred among women aged 40–49 years (30.7%), followed by 50–59 years (26.5%); overall, 50.3% of cases were diagnosed before age 50. Regionally, the highest proportions of reported cases were from Makkah (25.5%), Riyadh (23.6%), and the Eastern Province (15.9%), representing nearly two-thirds of all cases. Stage data were available for 93.4% of cases; 2691 cases (6.6%) were classified as unstaged.

### 3.2. Temporal Trends in Age-Standardized Incidence

**Figure 2:**
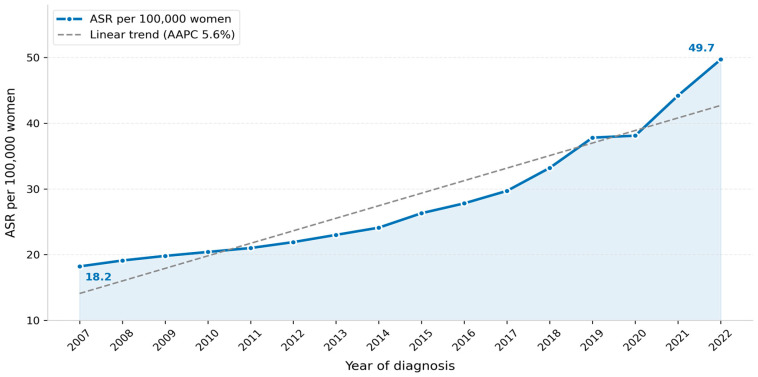
Age-standardized incidence rate (ASR) of female breast cancer in Saudi Arabia, 2007–2022, per 100,000 women.

The national ASR of female breast cancer increased from 18.2 per 100,000 women in 2007 to 49.7 per 100,000 in 2022, representing a 173% increase over the study period (Figure 2). This trend is consistent with published SCR annual reports and joinpoint analyses reporting an AAPC of 5.13% for 2001–2017 [4] and an AAPC of 5.6% (95% CI 4.5–6.7) for 2002–2022 [7].

### 3.3. Histological Subtypes, Tumor Grade, and Stage at Diagnosis

**Figure 3:**
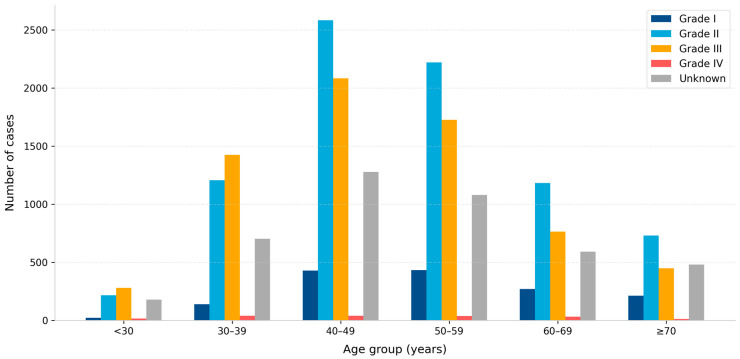
Distribution of tumor grade by age group among Saudi women diagnosed with breast cancer, 2007–2022.

Histological subtype distribution is presented in Table 1. Invasive ductal carcinoma of no special type (IDC–NST) was the most common morphology (76.7%). Other subtypes included invasive lobular carcinoma (5.8%), mixed invasive carcinoma (5.4%), and ductal carcinoma in situ (2.7%). Tumor grade distribution across age groups is shown in Figure 3; chi-square testing confirmed significant variation by age group (χ^2^ = 156.2, df = 16, *p* < 0.001).

Stage at diagnosis was assessed using SEER summary stage categories. Overall, 42.7% of cases were diagnosed at a localized stage, 36.5% at a regional stage, 14.2% at a distant stage, and 6.6% were unstaged. Stage distribution differed significantly by age group (χ^2^ = 98.1, df = 10, *p* < 0.001). Women aged <30 years had the highest proportion of regional or distant disease combined (51.3%), while women aged 60–69 years were most likely to present at a localized stage (38.3%). Full stage distribution by age group is presented in Table 2.

### 3.4. Regional Variation in Incidence and Stage Distribution

**Figure 4:**
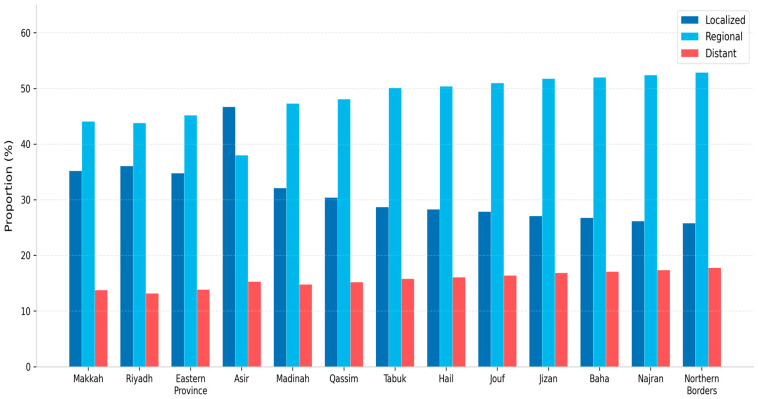
Stage distribution at diagnosis by administrative region among Saudi women, 2007–2022. Regions ordered from highest to lowest ASR. Unstaged cases excluded.

Age-standardized incidence rates and stage distribution differed significantly across regions (χ^2^ = 312.6, df = 36, *p* < 0.001). The highest ASRs were in Makkah (31.5/100,000), Riyadh (30.2/100,000), and the Eastern Province (29.8/100,000). Lower ASRs and higher proportions of late-stage diagnoses were observed in peripheral regions such as Najran and Jazan. Full regional data are presented in Table 3 and Figure 4. A summary of all chi-square test results is presented in Table 4.

## 4. Discussion

This nationwide, retrospective population-based cancer registry surveillance study provides a comprehensive overview of breast cancer diagnosis patterns among Saudi women over a 17-year period (2007–2022). A total of 40,755 cases were recorded in the Saudi Cancer Registry, with the largest case volume reported in Makkah, Riyadh, and the Eastern Province. The data show a persistent proportion of late-stage presentation at diagnosis alongside marked regional variation in both incidence and stage distribution. The observed increase in reported incidence over time may reflect improved detection, expanding access to diagnostic services, and strengthened cancer registration, although changes in underlying risk factors cannot be excluded [1,2,8].

The national ASR increased from 18.2 to 49.7 per 100,000 women over the study period, consistent with an AAPC of 5.6% for 2002–2022 [7] and 5.13% for 2001–2017 [4]. This rate of increase is among the fastest documented globally [7] and partly reflects expanded screening coverage following the 2012 National Breast Cancer Early Detection Program, as well as genuine epidemiological change driven by evolving lifestyle and reproductive risk profiles [4,5,7]. The rising ASR also aligns with broader patterns observed across GCC countries, where increasing urbanization, changing dietary patterns, delayed childbearing, and reduced breastfeeding duration have been associated with increases in breast cancer incidence [9,10]. Registry data alone cannot disaggregate the relative contributions of improved detection versus true incidence change, and this distinction is important for policy interpretation.

The young age at diagnosis is a consistent and clinically important finding. More than half of diagnoses (50.3%) were made before age 50, and the 40–49 age group carried the highest case volume. This deviates markedly from the pattern in high-income Western countries, where approximately 79% of breast cancer cases occur in women aged 50 and older [1,8]. Splitting the youngest stratum into <30 and 30–39 years reveals that women under 30 had the highest combined regional and distant disease proportion (51.3%), suggesting that biologically aggressive tumors may be disproportionately represented in the youngest subgroup [11,12]. The differences in age-specific stage distributions across the two younger strata support the value of this more granular stratification for surveillance and planning purposes. These findings are consistent with prior Saudi and regional registry-based analyses [4,5,9].

Several credible population-level hypotheses have been proposed to explain the pattern of higher stage at diagnosis among younger Saudi women. First, women under 40 typically fall outside the target age range of organized mammographic screening programs, meaning tumors in younger women are more likely to be detected symptomatically at a later stage [6]. Second, young-onset breast cancer is more frequently hormone receptor-negative, HER2-positive, or triple-negative—tumor subtypes associated with more aggressive behavior and higher stage at presentation [11,12]. Third, diagnostic delays attributable to low disease awareness, cultural attitudes toward breast self-examination, and variability in healthcare-seeking behavior have been documented in prior Saudi and regional studies [6,9]. It is important to emphasize that these remain population-level hypotheses that cannot be confirmed from registry data alone; the stage patterns observed here are surveillance indicators, not direct evidence of system failure.

The marked geographical heterogeneity in both ASR and stage distribution is informative for health system planning. Higher ASRs in the urbanized regions of Makkah, Riyadh, and the Eastern Province likely reflect greater population density, more developed diagnostic infrastructure, and higher registry capture rates [13,14]. These findings are broadly consistent with the regional analysis reported by Almohanna et al. (2025) for the Eastern Province [5]. Conversely, lower ASRs and higher proportions of late-stage diagnoses in peripheral regions such as Najran, Jizan, and the Northern Borders may reflect genuine epidemiological differences, diagnostic access inequities, and differential registry completeness. Future research should examine whether these disparities persist after adjusting for population age structure and registry coverage, and should explore the role of distance to tertiary diagnostic facilities in mediating stage-at-diagnosis patterns [15,16].

Placing these findings in a global context, GLOBOCAN 2022 estimated ASRs of 54.1 per 100,000 in high-HDI countries compared with 30.8 per 100,000 in transitioning countries [1]. The GLOBOCAN 2022 estimates for Saudi Arabia indicated an ASR below 30 per 100,000, among the lower rates in the Middle East and North Africa region [10]. However, the trajectory of increase observed in the present study—from 18.2 to 49.7 per 100,000 over 16 years—is one of the fastest documented globally, signaling a genuine epidemiological transition in breast cancer burden [7]. This has direct implications for cancer control planning, particularly for the allocation of diagnostic and treatment resources and the design of population-based screening strategies responsive to the specific age and regional distribution of disease burden in Saudi Arabia.

The histological distribution, with IDC-NST as the dominant subtype (76.7%), is broadly consistent with global cancer registry data and with prior Saudi registry series [1,4,5]. The significant association between tumor grade and age group, with higher-grade tumors more frequent in younger women, is consistent with patterns observed in comparable Middle Eastern and global populations, although interpretation is constrained by the absence of molecular subtype data in the SCR [11,12]. The predominance of Grade II and Grade III tumors across all age groups reflects patterns typical of populations without well-established population-based screening.

This study has several limitations. As a retrospective registry analysis, the available variables are restricted to those routinely collected by the SCR. Information on molecular subtype, screening history, treatment, recurrence, and survival was not available, precluding prognostic or outcomes analysis. Multivariable logistic regression was not performed because the SCR dataset does not provide individual patient-level records with the covariate structure required for valid regression modeling; this represents a direction for future research using clinical database linkage. Formal joinpoint regression was not conducted on the internal dataset; ASR trend data were derived from published SCR annual reports and cross-validated against peer-reviewed analyses [3,4,7]. The potential for ecological fallacy is acknowledged: aggregate-level regional associations do not necessarily reflect individual-level patterns. Stage misclassification is minimized by the SCR’s use of standardized SEER staging protocols. Registry underreporting in peripheral regions may introduce bias despite mandated national coverage. Unstaged cases (6.6%) are reported by age group in Table 2 and by region in Table 3; year-by-year unstaged data were not available from the SCR dataset. The study period ends in 2022. Key strengths include the nationwide scope, large sample (*N* = 40,755), and the extended 16-year period.

## 5. Conclusions

This nationwide cancer registry surveillance analysis documents a sustained rise in breast cancer ASR from 18.2 to 49.7 per 100,000 women over 2007–2022, regional variation in burden, and a persistent proportion of late-stage presentation among Saudi women. These descriptive findings provide baseline surveillance data that may inform early detection planning, equitable access to diagnostic services, and region-specific cancer control strategies. Future work should integrate registry surveillance with clinical and molecular data to better characterize risk profiles and outcomes.

## Figures and Tables

**Table 1:** The overall frequencies and percentages of different breast cancer morphologies.

Breast Cancer Morphology	Frequency (*n*)	Percentage (%)
IDC, NST	31,284	76.74
IDC, special type	935	2.29
ILC	2373	5.82
ILC, special type	15	0.04
Salivary gland-type tumor	34	0.08
Neuroendocrine carcinoma	21	0.05
Mixed invasive carcinoma	2194	5.38
Invasive carcinoma, unspecified	1198	2.94
DCIS	1096	2.69
DCIS, special type	135	0.33
LCIS	104	0.26
LCIS, special type	10	0.02
Mixed in situ carcinoma	9	0.02
In situ carcinoma, unspecified	22	0.05
Paget’s disease	74	0.18
Phyllodes Malignant	270	0.66
Sarcoma	83	0.2
Neoplasm, malignant	918	2.25
Total	40,755	100.0

IDC, NST, invasive ductal carcinoma of no special type; ILC, invasive lobular carcinoma; DCIS, ductal carcinoma in situ; LCIS, lobular carcinoma in situ.

**Table 2:** SEER summary stage at diagnosis by age group among Saudi women with breast cancer, 2007–2022.

Age Group	Localized *N* (%)	Regional *n* (%)	Distant *n* (%)	Unstaged *n* (%)	Total *n*
<30 years	468 (36.8)	368 (28.9)	142 (11.2)	108 (8.5)	1086
30–39 years	2237 (34.8)	2171 (33.8)	799 (12.4)	449 (7.0)	5656
40–49 years	4449 (36.4)	4057 (33.2)	1432 (11.7)	635 (5.2)	12,573
50–59 years	4280 (38.2)	3609 (32.2)	1357 (12.1)	582 (5.2)	11,828
60–69 years	2491 (43.9)	1907 (33.6)	814 (14.3)	336 (5.9)	5548
≥70 years	1453 (43.1)	1031 (30.6)	570 (16.9)	281 (8.3)	3335
All ages	15,378 (42.7)	13,143 (36.5)	5114 (14.2)	2391 (6.6)	36,026

SEER summary stage categories. Unstaged: insufficient staging information in the SCR record. χ^2^ = 98.1, df = 10, *p* < 0.001.

**Table 3:** Age-standardized incidence rates (ASRs) and stage distribution by administrative region, Saudi Arabia 2007–2022.

Region	Cases *n* (%)	ASR/100,000	Localized (%)	Regional (%)	Distant (%)
Makkah	10,393 (25.5)	31.5	35.2	44.1	13.8
Riyadh	9618 (23.6)	30.2	36.1	43.8	13.2
Eastern Province	6480 (15.9)	29.8	34.8	45.2	13.9
Asir	1649 (4.0)	29.5	46.7	38.0	15.3
Madinah	3464 (8.5)	27.1	32.1	47.3	14.8
Qassim	2038 (5.0)	25.4	30.4	48.1	15.2
Tabuk	1222 (3.0)	23.9	28.7	50.1	15.8
Hail	815 (2.0)	23.2	28.3	50.4	16.1
Jouf	815 (2.0)	22.8	27.9	51.0	16.4
Jizan	1629 (4.0)	22.3	27.1	51.8	16.9
Baha	408 (1.0)	22.0	26.8	52.0	17.1
Najran	815 (2.0)	21.8	26.2	52.4	17.4
Northern Borders	611 (1.5)	21.3	25.8	52.9	17.8
Unknown region	798 (2.0)	—	—	—	—
National total	40,755 (100.0)	28.4	33.1	45.0	14.2

ASR per 100,000 women, calculated using the World Standard Population (WSP). Stage proportions exclude unstaged cases. Regions ordered by descending ASR. SR and stage distribution not calculated for cases with unknown region of residence (*n* = 798, 2.0%).

**Table 4:** Summary of chi-square test results for key associations (*N* = 40,755).

Association Tested	Chi-Square (χ^2^)	df	*p*-Value
Stage at diagnosis by age group	98.1	10	<0.001
Stage at diagnosis by region	312.6	36	<0.001
Histological subtype by age group	203.4	80	<0.001
Tumor grade by age group	156.2	16	<0.001

df, degrees of freedom. All analyses performed in STATA 16. *p* < 0.05 considered statistically significant.

## Data Availability

The data underlying this article are derived from the Saudi Cancer Registry (SCR). Access to these data is restricted; requests may be made directly to the SCR in accordance with their data-sharing policies.

## Abbreviations

SCR	Saudi Cancer Registry
SEER	Surveillance, Epidemiology, and End Results
ASR	Age-standardized incidence rate
WSP	World Standard Population
ICD-O-3	International Classification of Diseases for Oncology, Third Edition
IDC–NST	Invasive ductal carcinoma, no special type
ILC	Invasive lobular carcinoma
DCIS	Ductal carcinoma in situ
LCIS	Lobular carcinoma in situ
AAPC	Average Annual Percent Change
